# Supplementary material for: Cell-body curvature reduces stall frequency to enhance Vibrio cholerae swimming and chemotaxis through hydrogels
Source: Commun Biol. 2026 Sep 8;9:1180. doi: 10.1038/s42003-026-10883-9 (PMC13554207; doi:10.1038/s42003-026-10883-9)
Supplement: Supplementary file 1 [file 42003_2026_10883_MOESM1_ESM.pdf]

SUPPLEMENTARY INFORMATION

Malik et al., "Cell-body curvature reduces stall frequency to enhance *Vibrio cholerae* swimming and chemotaxis through hydrogels"

Supplementary Discussion: on the choice of PVP K90 and soft agar hydrogels as simple mucus-mimicking media.

Mucus thickness, composition, and viscoelastic properties differ widely, from the thin layer protecting our eyes to the several hundred-micron thick multilayered lining in our colon[1–4]. Mucus structure at the bacterial scale ranges from an almost purely viscous solution to a highly confining hydrogel network with sub-micron pore sizes[5] and scale-dependent rheological properties[3,6,7]. The mucus of interest here is the loosely attached small-intestine mucus, as it is the barrier that *V. cholerae* must cross to reach its infection sites deep into small-intestine crypts[8]. Small-intestine mucus contains gelling mucins MUC2 and displays a mesh structure. In the healthy human small intestine, shear moduli were recently reported just under 10 Pa[9], in the lower range of the typical 1-100 Pa span reported along the whole intestine in various animal models[10]. Recent estimates of median pore sizes of canine small-intestinal mucus (using Cryo-SEM) are close to 2-3.5 µm. The bulk viscosity of human or pig small-intestine mucus is within 60-5,000 cP[7], with shear-thinning properties.
Agarose hydrogels display a nonlinear relationship between percentage and average pore size, with a widening distribution at lower percentages (e.g. see Pernodet et al.[11] Fig. 5). The exact porosity of agar, a lower purity polymer of agarose of ill-characterized molecular weight (which can influence pore size further[12]), is therefore hard to predict. Estimates are in the range of pores close to bacterial size; Licata et al.[13] estimated an average pore size for 0.25% agar in the 0.7-4.8 µm range. The elastic component (shear modulus) of 0.2% agar is expected to be close to that of 0.2% agarose (recently measured at 260 Pa[14]).
Solutions of polyvinyl pyrrolidone (PVP), a linear unbranched polymer with different molecular weights available, are widely used to study the relationship between viscosity and bacterial motility[15–17], but do not recapitulate the mesh structure of intestinal mucus. PVP K90 (average molecular weight of 360 kDa) has been demonstrated to display non-Newtonian shear thinning rheological properties down to microbial motility scale[16]. Solutions up to 40 cP can be easily and repeatability handled in the laboratory. We therefore used here mostly 0.2% agar hydrogels, as well as PVP K90, as two rheological extremes of mucus-mimicking media. PVP K90 lacks the hydrogel structure but may reflect the viscous non-Newtonian non-gelling (very low shear modulus) mucus while agar hydrogels may reflect the hydrogel mesh structure but with a higher shear modulus (lower deformability) than at-rest small-intestine mucus. We then leveraged an in-silico model to extend our experimental agar results to mesh with realistic small-intestine shear moduli.

Supplementary Tables

strain	Total number of motile trajecto-ries	# of analyzed trajecto-ries	Cumul. duration (s)	Motile speed (µm/s)	Turning freq. (s^{-1})	Forward duration (s)	Backward duration (s)
wt	6,897	3,554	9,547	84.8 ±1.6	1.30 ±0.13 (12,921)	0.46 ±0.03 (2,810)	0.156 ±0.002 (3,790)
Δ*crvAB*	5,305	2,796	7,999	81.5 ±1.0	1.32 ±0.06 (10,708)	0.47 ±0.02 (3,336)	0.156 ±0.007 (4,500)

SI Table 1 – **Detailed dataset of wt and Δ*crvAB* strains' random swimming motility across three replicates in buffer.** Displayed values are cumulative sum (total number of trajectories and analyzed trajectories) or averages ± standard deviations across independent replicates. The total numbers of events across replicates are indicated in parentheses. Bacteria are deemed motile if their average swimming speed exceeds 15 µm/s. Trajectories are analyzed when exceeding 0.8 s in duration and 15 µm/s in average swimming speed. Forward and backward runs were identified by their bordering turning angles (see Methods). The detailed data here corresponds to analysis results displayed in Figure 1.

Strain	Total number (duration) of trajectories	Number (duration) of analyzed trajectories	Swim extent (µm)	Swim phase v_d (µm/s)	Stall entrance freq. (s^{-1})	Stall escape freq. (s^{-1})	Stall duration (s)	RMS swim speed (µm/s)
wt	32,577 (290,991 s)	28,984 (288,256 s)	21.6 ±2.2SD	0.97 ±.3SD	0.70 ±.05SD	0.79 ±.09SD	1.28 ±.16SD	33 ±3SD
Δ*crvAB*	23,113 (239,891 s)	20,937 (238,238 s)	16.1 ±1.5SD	0.62 ±.2SD	0.88 ±.06SD	0.80 ±.10SD	1.27 ±.16SD	32 ±2SD
p-value			2.10^{-5}	0.008	5.10^{-6}	0.35 (n.s.)	0.32 (n.s.)	0.3 (n.s.)

SI Table 2 – **Detailed dataset of wt and Δ*crvAB* strains' swim-stall motility and chemotaxis parameters across seven replicates in 0.19-0.21% agar.** Displayed values are cumulative sum (total number of trajectories and analyzed trajectories) or averages ± standard deviations across independent replicates. The analyzed trajectories are all trajectories with a duration of or higher than 1 second. The detailed data here corresponds to analysis results displayed in Figure 3. Indicated p-values are from a two-sided paired t-test with unknown variance on each column's measurement, between wt and Δ*crvAB*, over the n=7 replicates.

Conditions	Shape	# of swim phases	Exponential decay length estimate λ (µm)	Ratio of exponential lengths $\lambda_{curved}/\lambda_{straight}$
Experimental (agar ~.20%)	Curved (wt)	70,545	28 ±1.7 (SD, n=7)	1.26 ±0.10 (SD, n=7)
	Straight (Δ*crvAB*)	60,727	22 ±1.7 (SD, n=7)	
In silico (~100 Pa)	Curved	1000	100 ±6 (95%CI)	1.25
	Straight	1000	80 ±5 (95%CI)	

SI Table 3 – **Summary of exponential decay length estimates from experimental and in-silico survival distributions of swimming phase extents.** In experimental data, the decay length is estimated for each phenotype on each experimental replicate and the noise reflects the standard deviation over the n=7 replicates. In in-silico data, the slope is estimated for each shape over its full distribution (for n=1000 simulations) and the noise reflects the 95% confidence intervals. The estimates and their 95% CI were obtained using the expfit function (MATLAB R2023a). The in-silico parameters were curvatures of 0 and 0.71 μm^{-1} for the straight and curved cell shapes respectively, in a mesh with a shear modulus of 95.2 Pa. The detailed data here corresponds to analysis results displayed in Figure 4a.

Supplementary Figures

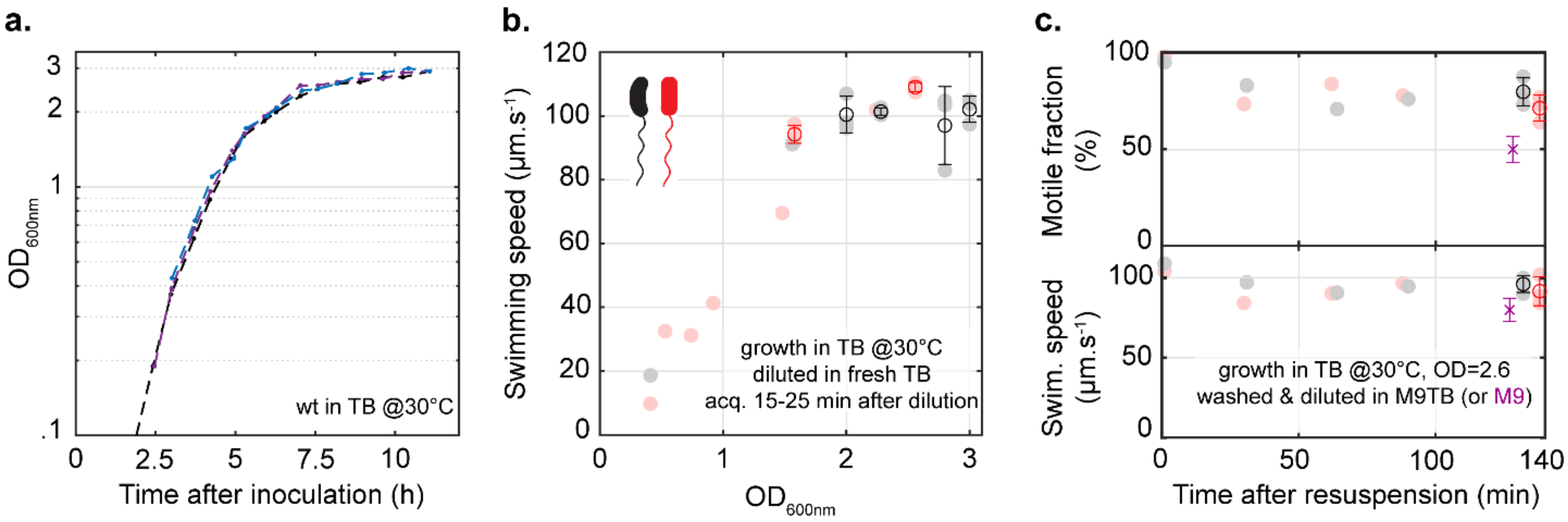

SI Figure 1 – **Growth and acquisition conditions ensuring stable motility parameters over acquisition times.** **(a)** Growth curve of wt strain in TB at 30°C 250 rpm, measured via optical density at 600nm (OD_{600nm}) over three independent experiments. **(b)** Swimming speed of motile fraction as a function of OD_{600nm}. The culture is diluted to an approximate OD_{600nm}=0.005 in fresh TB, then a 3D-tracking acquisition is made after 15-25 min to allow for adaptation. Points represent technical replicates over a single experiment. **(c)** Motile fraction and swimming speed over time, in final growth and acquisition conditions chosen (except for OD_{600nm}=2.6 instead of 2.2-2.4). The motility parameters are stable beyond the maximum duration after dilution accepted in further experiments (120 minutes). Points represent technical replicates over a single experiment. M9 salts + 0.01% TB (M9TB) was chosen as motility medium instead of M9 salts alone (purple point, wt strain) due to a clear decrease in motile fraction in the latter.

SI Figure 2 – **Further navigation properties in chemotaxis experiments in simple buffer or in PVP K90 5% (~38 cP).** **(a)** Average bacterial swimming speed distributions and averages and standard deviations over n=10 replicates. **(b)** Average swimming speed and root-mean-squared (r.m.s.) swimming speed of wt against Δ*crvAB* showing a small but repeatable increase of swimming speed for the curved-cell shape relative to the straight-cell one (on average 4.2 (±0.6 95% CI)%). **(c)** Average bacterial swimming speed distributions. **(d)** Drift velocity per RMS swimming speed bin, each bin determined to contain 10% of the total population. Error bars represent the 95% confidence intervals.

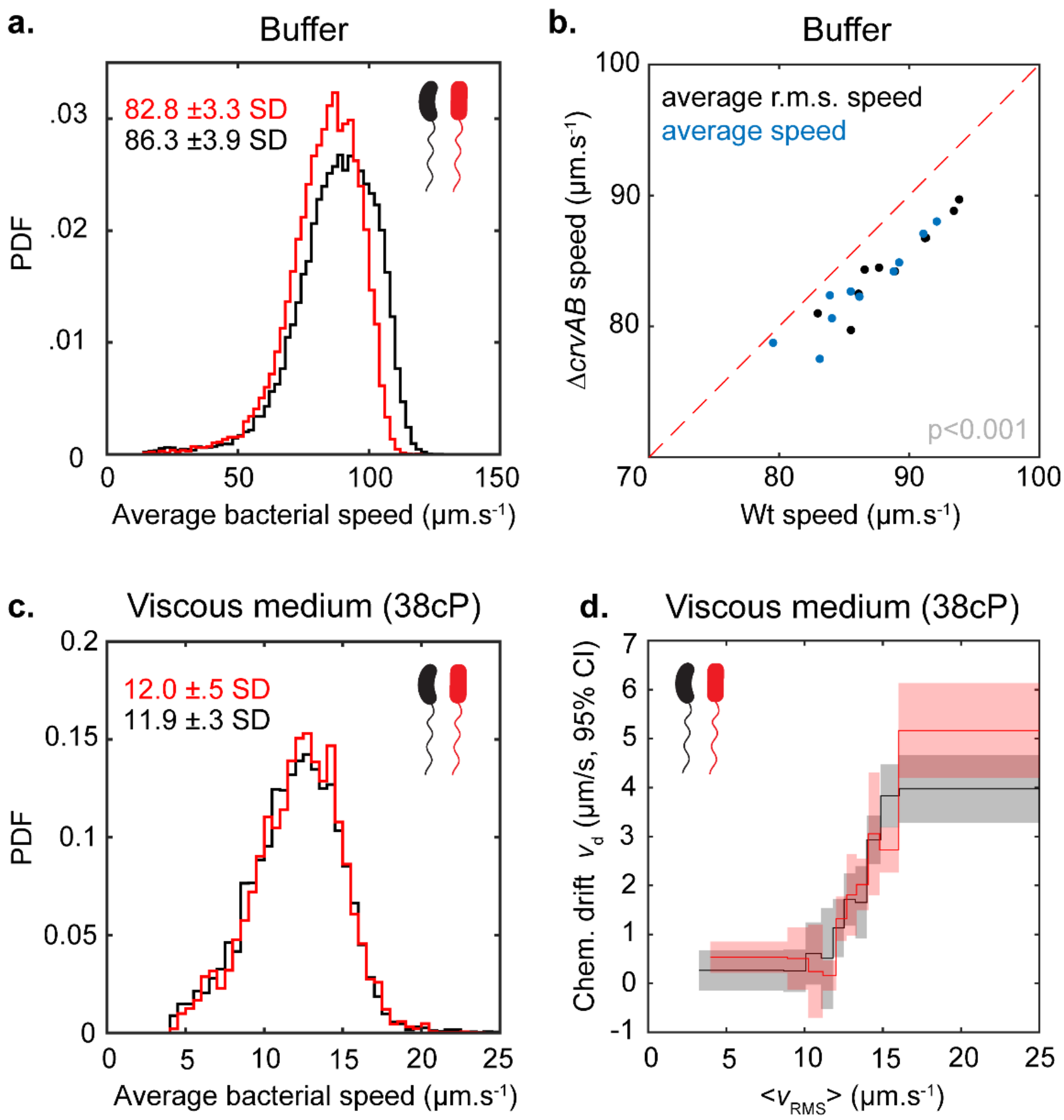

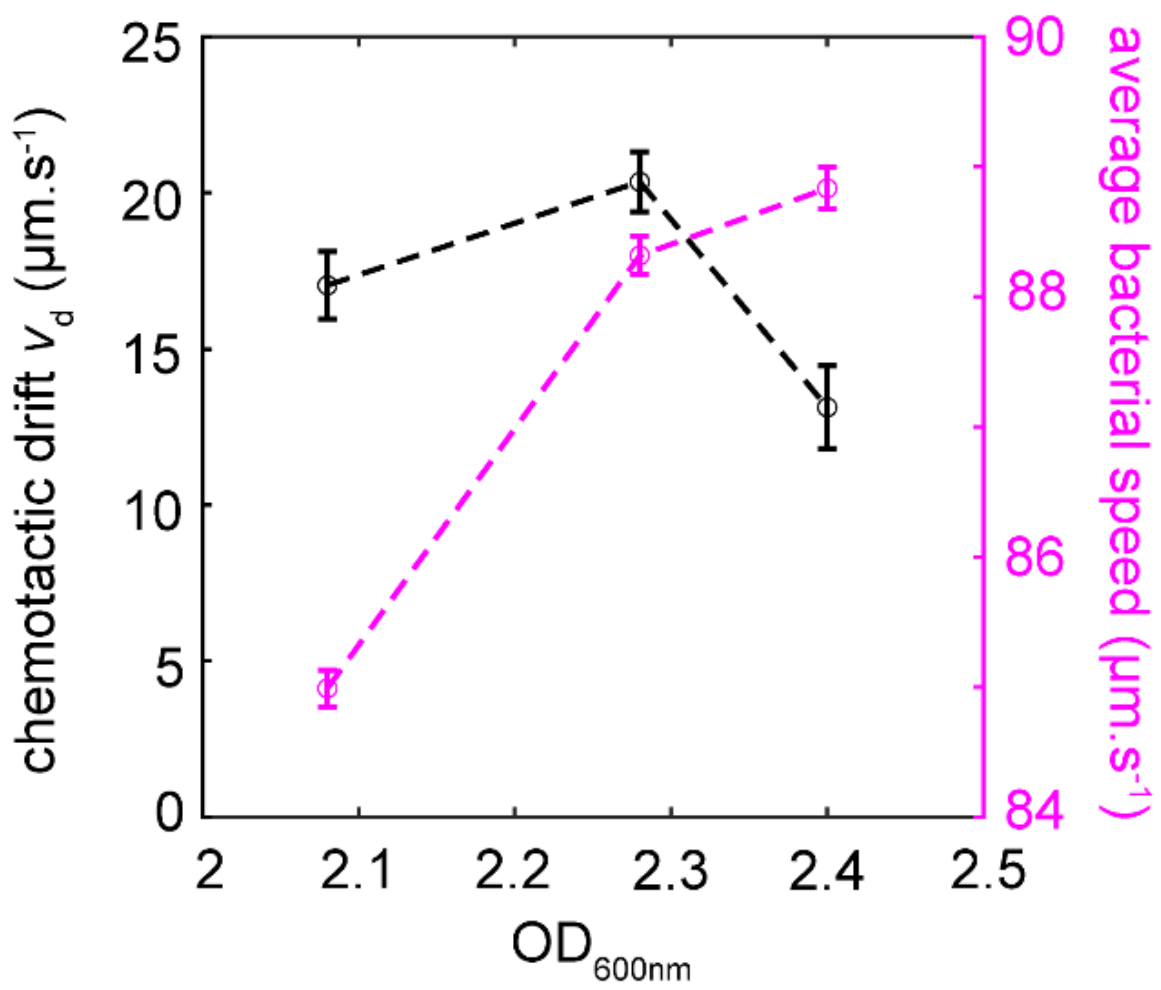

SI Figure 3 – **Chemotactic drift variability in buffer.** Chemotactic drift (black, left) and average swimming speed (magenta, right) for bacterial populations from one wt bacterial culture probed over three different OD values, following the same protocol as for regular chemotaxis experiment in buffer. Error bars represent the standard deviation between three successive technical replicates. While OD=2.2-2.4 was selected to ensure stable and high motile fractions (see SI Figure 1), chemotactic drifts showed a decrease as the OD increased. This imposed our strict requirement on identical OD (±0.01) within the same experiment to compare the strains (see Methods) and probably underlines the observed dispersed performances between experiments (Figure 2b).

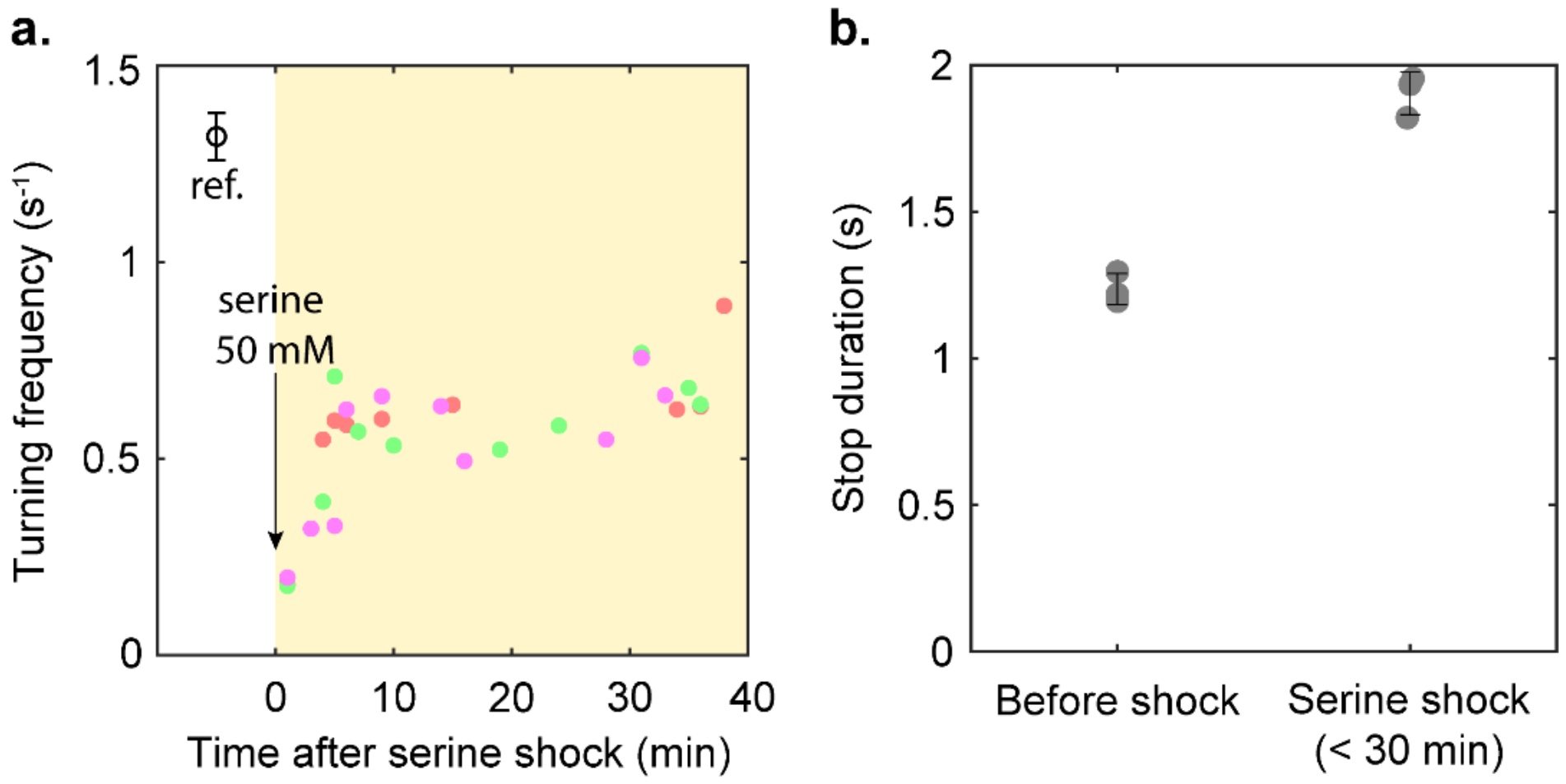

SI Figure 4 – **Data supporting turning as the primary mechanism for escaping stalls in hydrogels. (a)** Turning frequency of wt strain in M9TB after sudden exposure to 50 mM L-serine (so-called serine shock). **(b)** Stop durations of wt in soft agar in a chemotactic chamber under typical assay conditions (200 µM/mm serine gradient in M9TB, "before shock"), then after replacement of the reservoirs' content with M9TB + 50mM serine ("serine shock", within 30 min).

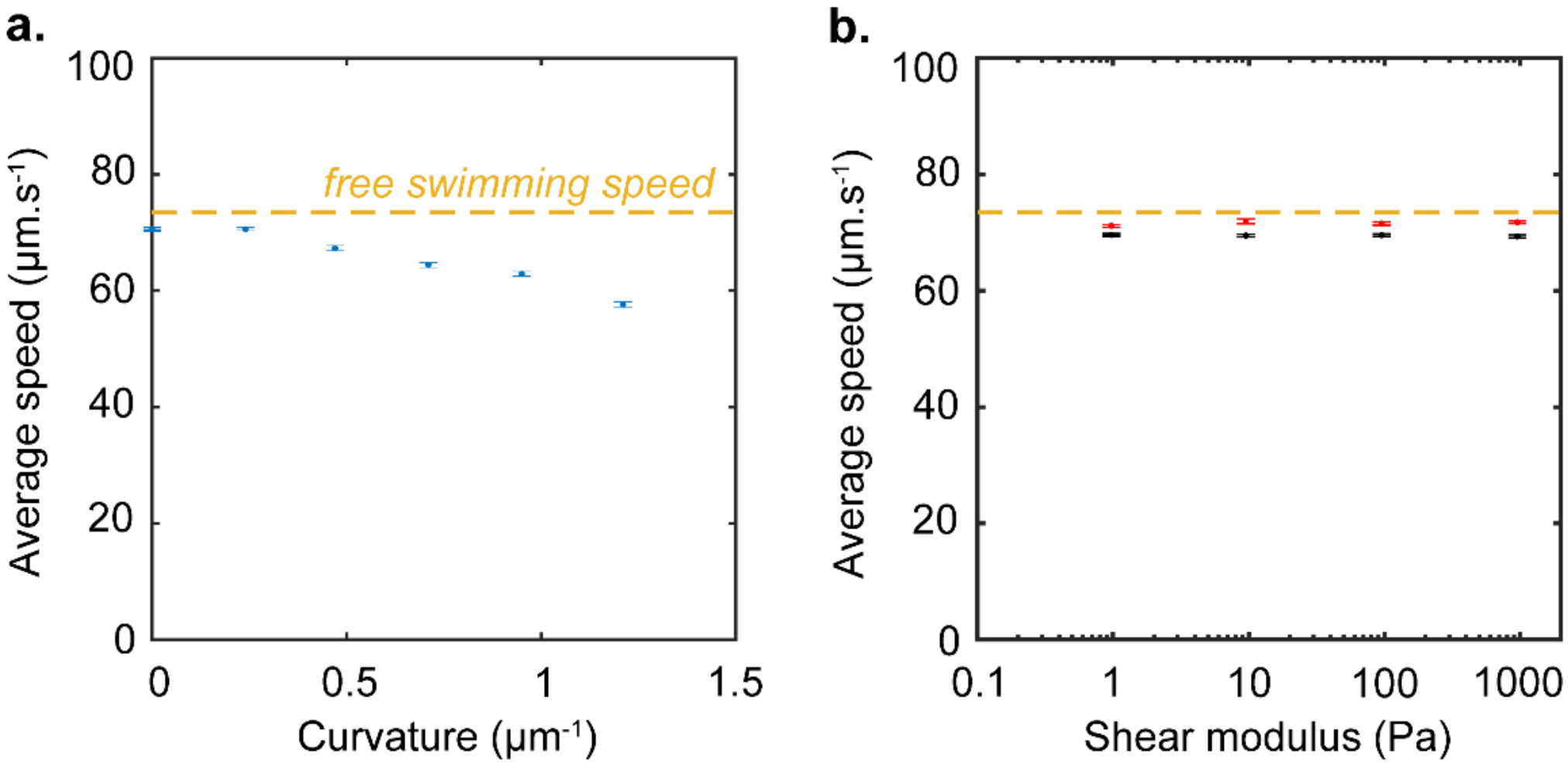

SI Figure 5 – **Average swimming speeds of "bacteria" in in-silico coarse-grained molecular dynamics simulations.** The average speeds and their 95% confidence intervals are represented over either **(a)** the curvature range tested or **(b)** over the range of stiffnesses tested for both straight (red) and curved (black) "bacteria".

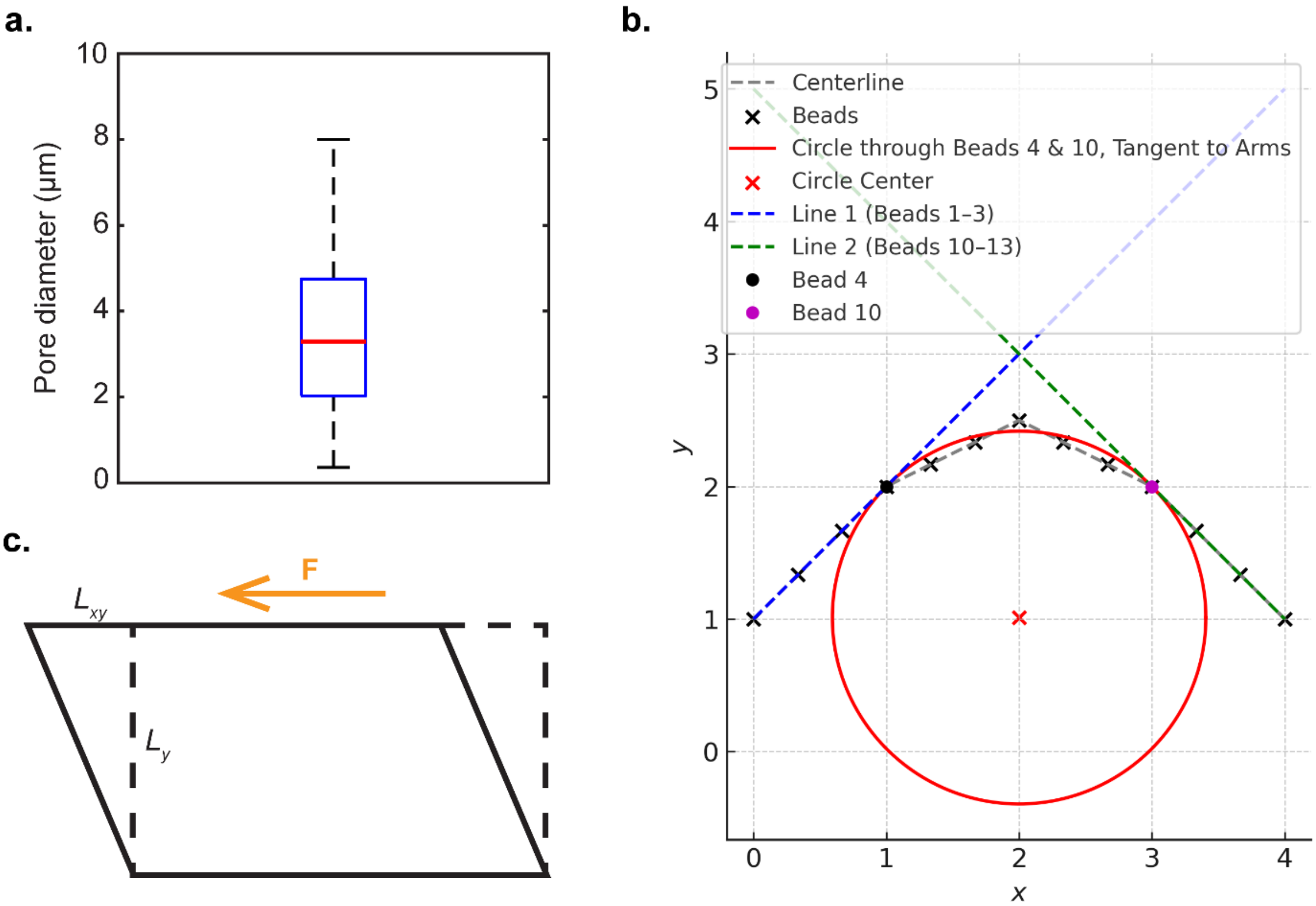

SI Figure 6 **– Details of parameters in our in-silico coarse-grained molecular dynamics model. (a)** Pore size distribution with an average pore size of 3.49 µm (±1.79 µm SD). This falls within estimated range of mean pore size for soft (0.25%) agarose hydrogels of 0.7-4.8 µm and close to recent estimates of median pore sizes of canine small-intestinal mucus (using Cryo-SEM) of 2-3.5 µm. **(b)** Estimating in-silico bacteria curvature (complementary illustration for Methods). **(c)** Measurement of in-silico mesh's shear modulus (complementary illustration for Methods).

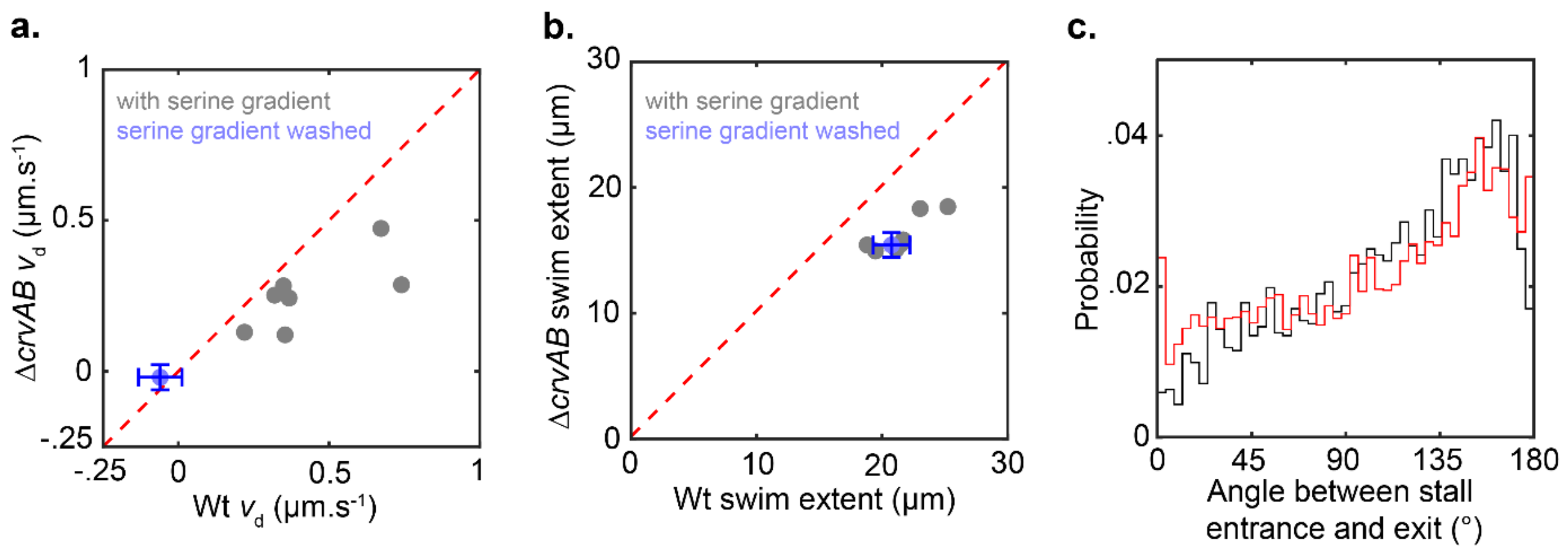

SI Figure 7 – **The curvature-induced improvement in swim extents (i.e. distance travelled between two stalls) is a direct effect of curvature on motility in soft hydrogels, not chemotaxis alone.** A chemotactic chamber was prepared as for regular chemotaxis experiment in 0.2% agar with a 200 µM/mm serine gradient, to allow bacteria to enter the soft agar. Then, the gradient was very gently washed away as to not damage the agar, by replacing the reservoirs' contents with fresh motility medium without serine every 10 minutes, 3-4 times in a row. **(a)** Chemotactic drift of analyzed trajectories in chamber with washed gradient (blue) compared to the chemotactic dataset used in Figure 3 (black). **(b)** Regardless of the absence of chemotactic response, the swim extent in chambers with washed gradient (blue) remained higher in wt compared to ΔcrvAB, as in the chemotactic dataset used in Figure 3 (black). In panel a and b, error bars reflect 95% confidence intervals estimated by a jackknife resampling procedure consisting of dividing the data into subsets of 200 trajectories and computing the SEM drift obtained for different subsets. The dotted red reference line marks identical performance of both phenotypes. This confirms that cell-body curvature directly influences motility in the agar mesh, not only chemotactic performance. **(c)** The angle between the entrance direction and the exit direction of stall phases in absence of gradient is similar to that in gradient (Figure 3g), highlighting further that the escape mechanism too is likely not dependent on chemotaxis.

SI References

1. Hormel, T.T., Bhattacharjee, T., Pitenis, A.A., Urueña, J.M., Sawyer, W.G., and Angelini, T.E. (2017). A Confocal Fluorescence Microscopy Method for Measuring Mucous Layer Growth on Living Corneal Epithelia. Biotribology *11*, 73–76. https://doi.org/10.1016/j.biotri.2017.04.004
2. Johansson, M.E.V., Phillipson, M., Petersson, J., Velcich, A., Holm, L., and Hansson, G.C. (2008). The inner of the two Muc2 mucin-dependent mucus layers in colon is devoid of bacteria. Proceedings of the National Academy of Sciences of the United States of America, 6. https://doi.org/10.4161/gmic.1.1.10470
3. Jory, M., Bellouma, K., Blanc, C., Casanellas, L., Petit, A., Reynaud, P., Vernisse, C., Vachier, I., Bourdin, A., and Massiera, G. (2019). Mucus Microrheology Measured on Human Bronchial Epithelium Culture. Frontiers in Physics *7*. https://doi.org/10.3389/fphy.2019.00019
4. Atuma, C., Strugala, V., Allen, A., and Holm, L. (2001). The adherent gastrointestinal mucus gel layer: thickness and physical state in vivo. American Journal of Physiology-Gastrointestinal and Liver Physiology *280*, G922–G929. https://doi.org/10.1152/ajpgi.2001.280.5.G922
5. Bansil, R., and Turner, B.S. (2018). The biology of mucus: Composition, synthesis and organization. Advanced Drug Delivery Reviews *124*, 3–15. https://doi.org/10.1016/j.addr.2017.09.023
6. Weigand, W.J., Messmore, A., Tu, J., Morales-Sanz, A., Blair, D.L., Deheyn, D.D., Urbach, J.S., and Robertson-Anderson, R.M. (2017). Active microrheology determines scale-dependent material properties of Chaetopterus mucus. PLOS ONE *12*, e0176732. https://doi.org/10.1371/journal.pone.0176732

7. Lai, S.K., Wang, Y.-Y., Wirtz, D., and Hanes, J. (2009). Micro- and macrorheology of mucus. Advanced Drug Delivery Reviews *61*, 86–100. https://doi.org/10.1016/j.addr.2008.09.012
8. Almagro-Moreno, S., Pruss, K., and Taylor, R.K. (2015). Intestinal Colonization Dynamics of Vibrio cholerae. PLOS Pathogens *11*, e1004787. https://doi.org/10.1371/journal.ppat.1004787
9. Kramer, C., Rulff, H., Ziegler, J.F., Mönch, P.W., Alzain, N., Addante, A., Kuppe, A., Timm, S., Schrade, P., Bischoff, P., et al. (2024). Ileal mucus viscoelastic properties differ in Crohn's disease. Mucosal Immunology *17*, 713–722. https://doi.org/10.1016/j.mucimm.2024.05.002
10. Sardelli, L., Pacheco, D.P., Ziccarelli, A., Tunesi, M., Caspani, O., Fusari, A., Briatico Vangosa, F., Giordano, C., and Petrini, P. (2019). Towards bioinspired *in vitro* models of intestinal mucus. RSC Adv. *9*, 15887–15899. https://doi.org/10.1039/C9RA02368B
11. Pernodet, N., Maaloum, M., and Tinland, B. (1997). Pore size of agarose gels by atomic force microscopy. Electrophoresis *18*, 55–58. https://doi.org/10.1002/elps.1150180111
12. Narayanan, J., Xiong, J.-Y., and Liu, X.-Y. (2006). Determination of agarose gel pore size: Absorbance measurements vis a vis other techniques. J. Phys.: Conf. Ser. *28*, 83–86. https://doi.org/10.1088/1742-6596/28/1/017
13. Licata, N.A., Mohari, B., Fuqua, C., and Setayeshgar, S. (2016). Diffusion of Bacterial Cells in Porous Media. Biophysical Journal *110*, 247–257. https://doi.org/10.1016/j.bpj.2015.09.035
14. Shahab, S., Kasra, M., and Dolatshahi-Pirouz, A. (2021). Design and construction of a novel measurement device for mechanical characterization of hydrogels: A case study. PLoS ONE *16*, e0247727. https://doi.org/10.1371/journal.pone.0247727
15. Grognot, M., Mittal, A., Mah'moud, M., and Taute, K.M. (2021). Vibrio cholerae Motility in Aquatic and Mucus-Mimicking Environments. Appl Environ Microbiol *87*, e01293-21. https://doi.org/10.1128/AEM.01293-21
16. Martinez, V.A., Schwarz-Linek, J., Reufer, M., Wilson, L.G., Morozov, A.N., and Poon, W.C.K. (2014). Flagellated bacterial motility in polymer solutions. Proceedings of the National Academy of Sciences *111*, 17771–17776. https://doi.org/10.1073/pnas.1415460111
17. Ferrero, R.L., and Lee, A. (1988). Motility of Campylobacter jejuni in a Viscous Environment: Comparison with Conventional Rod-shaped Bacteria. Microbiology *134*, 53–59. https://doi.org/10.1099/00221287-134-1-53
