# Supplementary material for: Cell-body curvature reduces stall frequency to enhance Vibrio cholerae swimming and chemotaxis through hydrogels
Source: Commun Biol. 2026 Sep 8;9:1180. doi: 10.1038/s42003-026-10883-9 (PMC13554207; doi:10.1038/s42003-026-10883-9)
Supplement: Supplementary file 2 [file 42003_2026_10883_MOESM2_ESM.pdf]

Description of Additional Supplementary Files:

File: Supplementary Data

Description: All numerical data in figures of main text are reported in an excel file, with one sheet per figure for Figure 1 and Figure 2 and Figure 4, or split per groups of panels for clarity for Figure 3 (Figure3a-b, Figure3c-f, Figure3g-h). The underlying distributions for Figure 4 are detailed in the two last excel sheets. Within each sheet, all datasets are grouped according to panels, with titles and axes (including dimensions) in bold

File: Supplementary Movie 1

Description: Molecular-dynamics simulation with a curved in-silico bacterium (blue, 0.71 µm-1) advancing in the mesh (yellow). The movie is centered on an event where the bacterium decelerates but avoids stalling by deflecting its trajectory. This movie corresponds to the caption in Figure 4c of Main Text.
