# Supplementary material for: Cell-body curvature reduces stall frequency to enhance Vibrio cholerae swimming and chemotaxis through hydrogels
Source: Commun Biol. 2026 Sep 8;9:1180. doi: 10.1038/s42003-026-10883-9 (PMC13554207; doi:10.1038/s42003-026-10883-9)
Supplement: Supplementary file 3 [file 42003_2026_10883_MOESM5_ESM.pdf]

nature portfolio

Corresponding author(s): Marianne Grognot

Last updated by author(s): Jun 13, 2026

Reporting Summary

Nature Portfolio wishes to improve the reproducibility of the work that we publish. This form provides structure for consistency and transparency in reporting. For further information on Nature Portfolio policies, see our Editorial Policies and the Editorial Policy Checklist.

Statistics

For all statistical analyses, confirm that the following items are present in the figure legend, table legend, main text, or Methods section.

n/a	Confirmed	
☐	☒	The exact sample size (*n*) for each experimental group/condition, given as a discrete number and unit of measurement
☐	☒	A statement on whether measurements were taken from distinct samples or whether the same sample was measured repeatedly
☐	☒	The statistical test(s) used AND whether they are one- or two-sided<br>*Only common tests should be described solely by name; describe more complex techniques in the Methods section.*
☒	☐	A description of all covariates tested
☒	☐	A description of any assumptions or corrections, such as tests of normality and adjustment for multiple comparisons
☐	☒	A full description of the statistical parameters including central tendency (e.g. means) or other basic estimates (e.g. regression coefficient) AND variation (e.g. standard deviation) or associated estimates of uncertainty (e.g. confidence intervals)
☐	☒	For null hypothesis testing, the test statistic (e.g. *F*, *t*, *r*) with confidence intervals, effect sizes, degrees of freedom and *P* value noted<br>*Give P values as exact values whenever suitable.*
☒	☐	For Bayesian analysis, information on the choice of priors and Markov chain Monte Carlo settings
☒	☐	For hierarchical and complex designs, identification of the appropriate level for tests and full reporting of outcomes
☒	☐	Estimates of effect sizes (e.g. Cohen's *d*, Pearson's *r*), indicating how they were calculated

Our web collection on statistics for biologists contains articles on many of the points above.

Software and code

Policy information about availability of computer code

Data collection	Video recordings of swimming bacteria were acquired using the proprietary software Camware provided online by the camera manufacturer (PCO). Bacterial 3D tracking code was done in MATLAB (2023a) using the custom code provided by Pr. K. M. Taute, initially published in Taute et al., Nat. Commun., 2015 (doi.org/10.1038/ncomms9776) and improved in Grognot et al, PNAS, 2023 (doi.org/10.1073/pnas.2301873120).
Data analysis	3D tracks analysis was done in MATLAB (2023a) as published in Grognot et al, PNAS, 2023 (doi.org/10.1073/pnas.2301873120) and Grognot et al, AEM, 2021 (doi.org/10.1128/AEM.01293-21) with only thresholding parameters modification, as described in the Methods.

For manuscripts utilizing custom algorithms or software that are central to the research but not yet described in published literature, software must be made available to editors and reviewers. We strongly encourage code deposition in a community repository (e.g. GitHub). See the Nature Portfolio guidelines for submitting code & software for further information.

Data

Policy information about availability of data

All manuscripts must include a data availability statement. This statement should provide the following information, where applicable:

- Accession codes, unique identifiers, or web links for publicly available datasets
- A description of any restrictions on data availability
- For clinical datasets or third party data, please ensure that the statement adheres to our policy

All the bacterial trajectories analysed in the main text (including core datasets as detailed in Table 1) will be made publicly available on the repository

RWTHPublications, as well as swim extent data 9both experimental and in silico) and in silico pore size distributions and supplementary movie, upon publication of this article. Any other data (e.g. experiments displayed only in SI) can be obtained from the corresponding author upon reasonable request.

Research involving human participants, their data, or biological material

Policy information about studies with human participants or human data. See also policy information about sex, gender (identity/presentation), and sexual orientation and race, ethnicity and racism.

Reporting on sex and gender	The study only used a model bacterial strain and its previously published mutant, in media not sourced from animals/ humans.
Reporting on race, ethnicity, or other socially relevant groupings	The study only used a model bacterial strain and its previously published mutant, in media not sourced from animals/ humans.
Population characteristics	n/a
Recruitment	n/a
Ethics oversight	Authorization covering work with both Vibrio cholerae strains was given by the German regional administration (Bezirksregierung Düsseldorf) under number 53.05-01-K-23-093.

Note that full information on the approval of the study protocol must also be provided in the manuscript.

Field-specific reporting

Please select the one below that is the best fit for your research. If you are not sure, read the appropriate sections before making your selection.

☒ Life sciences ☐ Behavioural & social sciences ☐ Ecological, evolutionary & environmental sciences

For a reference copy of the document with all sections, see nature.com/documents/nr-reporting-summary-flat.pdf

Life sciences study design

All studies must disclose on these points even when the disclosure is negative.

Sample size	Bacterial trajectories sample sizes were chosen to balance reasonable acquisition time, data storage capacities, chemoattractant gradient establishment time, and acceptable bacterial densities in the chemotaxis chamber as determined in previous works. Thousands of experimental trajectories were acquired in independent replicates. A number of 1,000 in silico trajectories was set per tested model parameter. Sample sizes were deemed sufficient as they yielded reproducible and statistically significant results.
Data exclusions	No trajectory data were excluded from analysis except for non-motile bacteria or bacterial tracks too short as defined in Methods. The main trajectory datasets used for analysis are detailed in SI Tables 1-2 and will be made available through RWTHPublications with a unique DOI upon acceptance. No in silico trajectory was rejected from the set 1,000 collected per tested condition.
Replication	All full attempts at replicating the core results are displayed in the manuscript (Fig. 2b). In agar one out of 7 experiments did not significantly replicate the core result.
Randomization	No randomization was applied.
Blinding	No blinding was applied.

Reporting for specific materials, systems and methods

We require information from authors about some types of materials, experimental systems and methods used in many studies. Here, indicate whether each material, system or method listed is relevant to your study. If you are not sure if a list item applies to your research, read the appropriate section before selecting a response.

Materials & experimental systems

n/a	Involved in the study
☒	☐ Antibodies
☒	☐ Eukaryotic cell lines
☒	☐ Palaeontology and archaeology
☒	☐ Animals and other organisms
☒	☐ Clinical data
☒	☐ Dual use research of concern
☒	☐ Plants

Methods

n/a	Involved in the study
☒	☐ ChIP-seq
☒	☐ Flow cytometry
☒	☐ MRI-based neuroimaging

Plants

Seed stocks	*Report on the source of all seed stocks or other plant material used. If applicable, state the seed stock centre and catalogue number. If plant specimens were collected from the field, describe the collection location, date and sampling procedures.*
Novel plant genotypes	*Describe the methods by which all novel plant genotypes were produced. This includes those generated by transgenic approaches, gene editing, chemical/radiation-based mutagenesis and hybridization. For transgenic lines, describe the transformation method, the number of independent lines analyzed and the generation upon which experiments were performed. For gene-edited lines, describe the editor used, the endogenous sequence targeted for editing, the targeting guide RNA sequence (if applicable) and how the editor was applied.*
Authentication	*Describe any authentication procedures for each seed stock used or novel genotype generated. Describe any experiments used to assess the effect of a mutation and, where applicable, how potential secondary effects (e.g. second site T-DNA insertions, mosiacism, off-target gene editing) were examined.*
